# Ultrasound-Guided Radiofrequency Ablation for Chronic Hip Pain Due to Osteoarthritis

**DOI:** 10.7759/cureus.53743

**Published:** 2024-02-06

**Authors:** Rodrigo Correia, Luís Oliveira, Inês Andrade, Miguel de Castro Correia, Eugénio Gonçalves, Andre Borges, Tiago Lopes, José Luís Carvalho

**Affiliations:** 1 Physical Medicine and Rehabilitation, Centro de Reabilitação do Norte, Vila Nova de Gaia, PRT; 2 Physical Medicine and Rehabilitation, Centro de Medicina de Reabilitação de Alcoitão, Lisboa, PRT; 3 Physical Medicine and Rehabilitation, Centro Hospitalar Vila Nova de Gaia/Espinho, Vila Nova de Gaia, PRT; 4 Physical Medicine and Rehabilitation, Centro Hospitalar de Trás-os-Montes e Alto Douro, Vila Real, PRT; 5 Intervention and Musculoskeletal Rehabilitation, Centro de Reabilitação do Norte, Vila Nova de Gaia, PRT

**Keywords:** ultrasound-guided interventional pain management, radiofrequency ablation (rfa), osteoarthritis of the hip, hip joint pain, chronic joint pain

## Abstract

Background and aims: Hip osteoarthritis (OA) has a prevalence of 2.9% in Portugal and is a related cause of pain and disability. A sufficient number of patients report these symptoms even after total hip arthroplasty (THA), while others are contraindicated to such surgery and suffer from uncontrolled pain. Percutaneous denervation of hip nerve branches using radiofrequency ablation (RFA) has emerged as a powerful therapeutic avenue to consider for patients with chronic hip pain.

Methods: Between January 2020 and March 2021, 26 patients with chronic hip pain received ultrasound-guided RFA with a pericapsular nerve group (PENG) block technique adaptation. Patients suffering from chronic hip pain for more than three months with radiographic evidence of osteoarthritis were included. A numeric rating scale (NRS) and pain medication reduction were defined as outcome variables assessed before treatment and at three-, six-, nine-, and 12-month follow-ups.

Results: All selected patients underwent the procedure. All the patients had hip osteoarthritis. Twelve-month follow-up data revealed a statistically significant decrease in the numeric rating scale. The mean NRS for pain was 2 after the procedure. Over 75% of patients reported >50% pain relief during the follow-up and 85% reduced pain medication consumption. No side effects were reported.

Conclusion: Hip sensory articular branch RFA is a treatment option with interesting outcomes for chronic hip pain, as demonstrated by our study.

## Introduction

Chronic hip pain is a prevalent and functionally limiting symptom that may be due to osteoarthritis (OA), rheumatoid arthritis, osteonecrosis, femoroacetabular impingement, infectious coxarthrosis, and post-total hip arthroplasty (THA) pain.

According to the Centers for Disease Control and Prevention, the lifetime risk for symptomatic hip OA is 18.5% for males and 28.6% for females [1]. While enough patients report these symptoms even after undergoing total hip arthroplasty (THA) surgery, others are contraindicated to such surgery and live with unmanageable pain. Thus, alternative therapeutic methods need to be sought. Percutaneous denervation of hip articular nerve branches using radiofrequency ablation (RFA) is emerging as a powerful therapeutic weapon to consider for patients with chronic hip joint pain [2-4].

In the majority of published studies, the fluoroscopic approach is the most commonly utilized method for radiofrequency ablation. However, Kapural et al. [5] conducted a study suggesting that a combined technique involving fluoroscopic guidance monitored with ultrasound may offer improved safety compared to the fluoroscopic approach alone. This combined approach aims to avoid potential damage to the femoral neurovascular bundle. It is important to note that the impact of incorporating ultrasound guidance on procedure safety and effectiveness is still uncertain, as stated by Cheney et al. [4]. Further research is needed to determine the potential benefits of ultrasound guidance in radiofrequency ablation procedures.

This retrospective study wants to evaluate the efficacy of ultrasound-guided radiofrequency ablation for chronic hip pain patients with osteoarthritis by analyzing the numeric rating scale (NRS) pain scale at three, six, nine, and 12 months post-intervention; oral medication consumption after intervention; and adverse effects or complications of the technique.

## Materials and methods

Between January 2020 and March 2021, 26 patients with chronic hip pain who received ultrasound-guided RFA of the articular branches of the femoral nerve (accountable for 75% of the sensitive afferents of the anterior hip capsule) in a rehabilitation center in Portugal were retrospectively analyzed using the hospital informatics software, SClínico®.

In this study, patients suffering from chronic hip pain for more than three months with radiographic osteoarthritis of the hip were included. Patients with femoroacetabular impingement, extrinsic source of hip pain, systemic sepsis, allergy to local anesthetics, and psychiatric illness, who are unable to comprehend pain scoring, and who underwent total hip replacement (THR) were excluded. A numeric rating scale (NRS) and reduced pain medications (yes or no) were used for outcome measurement before treatment and at three, six, nine, and 12 months after RFA.

The Tönnis scale (Table 1) was employed for the radiologic classification of hip osteoarthritis [6]. The reported inter- and intraobserver reliability of this scale varies from k = -0.02 to k = 0.76 [6]. The two physicians who conducted the interventions in this study were also the ones responsible for X-ray grading, as it was carried out during their consultations.

**Table 1:** Tönnis scale for hip osteoarthritis

Grade	Radiographic feature
0	No signs of osteoarthritis
1	Slight narrowing of joint space; slight lipping at joint margin; slight sclerosis of the femoral head or acetabulum
2	Small cysts in the femoral head or acetabulum; increasing narrowing of joint space; moderate to loss of sphericity of the femoral head
3	Large cysts; severe narrowing or obliteration of joint space; severe deformity of the femoral head; avascular necrosis

A pericapsular nerve group (PENG) block with ropivacaine and lidocaine was performed before the radiofrequency ablation technique to predict a positive or negative outcome.

The technique is performed with the patient in the supine position, and a curvilinear low-frequency ultrasound probe (2-5 Mhz) is placed on a transverse plane over the anterior superior iliac spine (ASIS). Once the ASIS is identified, the transducer is then aligned with the pubic ramus and rotated at approximately 45 degrees, parallel to the inguinal crease. The transducer is then slid medially along this axis until the anterior inferior iliac spine (AIIS), iliopubic eminence (IPE), and psoas tendon are clearly identified, serving as anatomic landmarks. Sliding the probe distally or gently tilting it caudally will expose the femoral head. Returning to the initial starting position, a standard 22-gauge 100 mm needle is inserted in-plane, from lateral to medial, in the plane between the AIIS and IPE. A 1:1 dilution of 5cc lidocaine plus 5cc ropivacaine is then deposited in this plane, lifting the psoas tendon.

**Figure 1:**
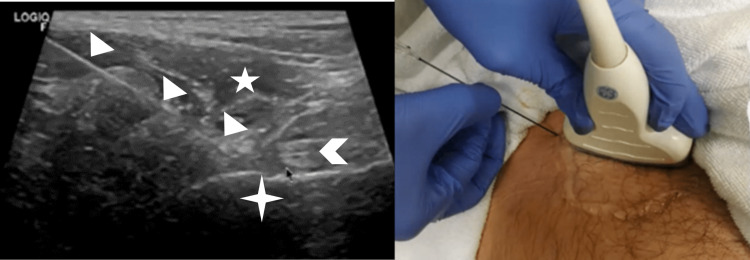
Ultrasound-guided radiofrequency ablation technique On the left is a transverse ultrasound image of the target to perform the radiofrequency ablation of femoral nerves. The patient is in prone position. Arrowhead: psoas tendon, triangular shape: needle, four-pointed star: pubic ramus, five-pointed star: iliacus muscle

After a positive diagnostic block (at least 50% pain reduction), the patients underwent RFA using a similar approach to the technique mentioned above, aiming for the majority of the articular rami of the femoral nerve. With the RFA needle in place (Unified™ EchoRF™ Boston Scientific, Marlborough, MA) (15 cm × 10 mm, 20 gauge), motor stimulation was performed to exclude the involvement of motor nerves (0.6-1.2 volts), and then, sensitive stimulation (0.3-0.6 volts) was performed with the purpose of reproducing the patient's symptoms. A dispersive plaque was placed in the contralateral limb of the patient. Once everything is checked, continuous radiofrequency is applied for two minutes to target the thermoablation temperature (80 degrees Celsius) (Figure 1). After the first lesion, three more lesions are made at three different sites along the pubic ramus, so that four lesions are made.

This study was approved by the ethics committee of the hospital where the study was conducted (CES number: 105/2023-1).

For statistical analysis, the Friedman analysis of variance (ANOVA) test was performed to compare the differences between NRS scores across time, when the normality assumption was not present (using Kolmogorov-Smirnov test and Shapiro-Wilk test, kurtosis, and graph visualization, especially histogram). All statistical analysis was performed using the Statistical Package for Social Sciences (SPSS) version 23.0 (IBM SPSS Statistics, Armonk, NY), and the results were considered significant if p<0.05.

## Results

A summarized description of the patients is presented in Table 2. Twenty-six patients were included in this study (11 females and 15 males, 42.3% and 57.7%, respectively). The median age was 63 years old. All the patients had primary hip osteoarthritis. Regarding radiologic osteoarthritis grading, 12 patients had a Tönnis grade 2 (34.5%), and 14 had a Tönnis grade 3 (48.3%).

**Table 2:** Patient description

Variable	Frequency (number)	Median	Total
Sex	Female: 11	-	26
	Male: 15		
Age (years)	-	63	-
Tönnis grading scale	Tönnis grade 2: 12	-	26
	Tönnis grade 3: 14		

About 85% of the patients had a reduction in analgesic consumption. No immediate or long-term complications were reported by the patients.

The median NRS scores are shown in Table 3. There was a significant reduction in NRS pain score, where NRS score at 0 months was 8 and post-procedure at three months was 2 (p<0.01), six months was 1 (p<0.05), nine months was 2 (p<0.05), and 12 months was 2 (p<0.05). During the 12-month follow-up, there was a significant reduction in hip pain (X^2^ (4)=84.454, p<0.01, Friedman ANOVA). The results of the post hoctest used (pairwise multiple comparisons tests) are presented in Table 4.

**Table 3:** Median numeric rating scales at 0, 3, 6, 9, and 12 months after the procedure RFA: radiofrequency ablation, NRS: numerical rating scale for pain, IQR: interquartile range

Evaluation (months)	0	3	6	9	12
NRS score median	8	1	0	2	3
NRS score IQR	7-9	0-3	0-2	0-3	0-4

**Table 4:** Pairwise method analysis

Group comparison	Pairwise method statistics (Dunn's test)	p-value
3 months pre-procedure	6.27	<0.01
6 months pre-procedure	6.18	<0.01
9 months pre-procedure	5.48	<0.01
12 months pre-procedure	4.65	<0.01

## Discussion

The prevalence of osteoarthritis in Portugal is 2.9% [7]. The risk of developing symptomatic osteoarthritis is 18.5% in males and 28.6% in females [1]. With the increasing quality of the healthcare systems and continuous advances in medical care, there is an increase in patients with multiple comorbidities. Therefore, some patients may present counterindications to performing a THR, and some patients may develop or maintain hip pain after total hip replacement.

In the available literature, there are no medical guidelines regarding patient selection for hip articular branch RFA [3-5,8-11]. Cheney et al. [4] reviewed patient selection where 89 patients had hip osteoarthritis, 15 patients had avascular necrosis, and four patients underwent THR [5]. In our study, 26 patients had hip osteoarthritis.

Among the available studies, ours had a higher number of evaluations in the follow-up (three, six, nine, and 12 months evaluation) and one of the largest sample sizes (26 patients) [3-5,8-15]. The studies reported a pain reduction of 30%-80%, where few demonstrated significant pain reduction for at least six months [8-15]. In our study, the mean pain reduction was over 75% for 12 months follow-up, which is very rewarding. In this study, we also evaluated analgesic consumption, and 85% of the patients had a reduction in their usage.

Ablation was performed only in the femoral sensory branches, given the characteristics of the pain presented at the time of evaluation. The pain reported was predominantly in the anterior, lateral, and superomedial quadrants of the hip. These quadrants are innervated by the sensory branches of the femoral nerve; therefore, we opted for the neuroablation of these branches in detriment of the sensory branches provided by the obturator nerve. We also add that the proper and effective realization of the sensory branches of the obturator nerve requires a technique guided by fluoroscopy; thus, they would have to be intervened in a suitable place for the purpose, which limits the performance of the technique in logistical terms. Thus, and with the results presented, there seems to be an important benefit of performing ultrasound-guided radiofrequency in patients with osteoarthritis of the hip, whose symptoms involve more of the aforementioned sensory quadrants.

Our study was performed only with an ultrasound guidance technique and showed similar or even superior outcomes when compared to the fluoroscopic guidance technique performed in other available studies [3-5,8-15], and no immediate or late complications were reported by the patients. This makes an interesting future approach since it is less expensive and has fewer logistics setbacks, no radiation exposure, and widespread availability of ultrasound equipment when compared to the fluoroscopic technique. This could augment the availability of this procedure among the physiatric offices.

Limitations

As a retrospective cohort study, our group cannot deny the existence of limitations. As it has less evidence of strength, our study can only demonstrate an association with an inherent selection and operator/observer bias.

Another limitation that we report is that, because this is a retrospective study, we have had limitations in the quantitative characterization of analgesic consumption, in which we only had access to the information found in the clinical process of the patients. Therefore, because the data were written in a non-standardized way, it was impossible for us to characterize it in the best way, so we chose to quantify only whether there was a reduction in the consumption of analgesics or not (NSAIDs or opiates).

Although we recognize the importance of functional questionnaires, it is very difficult to apply in daily practice. As such, this is a retrospective study, and it was not possible to obtain a functional score for our patients.

Further research is needed, particularly high-quality randomized controlled multicenter trials, that include functional analysis to create recommendation guidelines on this subject and, therefore, standardize this intervention approach as a chronic pain treatment in selected patients.

## Conclusions

Our study asserts the effectiveness of using ultrasound-guided femoral sensory branch RFA to ensure a prolonged and efficacious pain reduction in chronic hip pain reported by patients with hip osteoarthritis and chronic pain after THR. We believe that this is an interesting future approach because the reduction in pain was high and prolonged, making this an important, minimally invasive alternative option to total hip replacement. Additionally, we believe that there is an apparent advantage in using ultrasound guidance since it increases the accuracy of sensory branch nerve ablation.
